# Magnitude and Determinants of Patients at Risk of Developing Obstructive Sleep Apnea in a Non-Communicable Disease Clinic

**DOI:** 10.3390/medicina55070391

**Published:** 2019-07-20

**Authors:** Prakash Mathiyalagen, Venkatesh Govindasamy, Anandaraj Rajagopal, Kavita Vasudevan, Kalaipriya Gunasekaran, Dhananjay Yadav

**Affiliations:** 1 Department of Community Medicine, Indira Gandhi Medical College & Research Institute, Kathirkamam, Vazhudavur Road, Puducherry 605010, India; 2 Department of Community Medicine, Government Thiruvannamalai Medical College & Hospital, Thiruvannamalai 606604, India; 3 Department of Medical Biotechnology, Yeungnam University, Gyeongsan 38541, Korea

**Keywords:** obstructive sleep apnea, non-communicable disease, Modified Berlin Questionnaire, dyslipidemia, body mass index, waist circumference

## Abstract

*Background and Objective*: Obstructive sleep apnea (OSA) is a common chronic disorder worldwide, which can adversely affect the cardiovascular system among non-communicable disease (NCD) patients. It is underdiagnosed—or rather not diagnosed—in primary care settings due to the costly diagnostic techniques involved. This study aimed to assess the number of study participants at risk of developing OSA and to assess and quantify the risk factors associated with this disorder. *Materials and Methods*: A cross-sectional study was performed in an NCD clinic of a rural health training center, Karikalampakkam, Puducherry of South India from August 2018 to October 2018. A Modified Berlin Questionnaire (MBQ) was used to screen the study participants at risk for OSA. Four-hundred-and-seventy-three people aged 18 years and above were included in the study, using systematic random sampling. Respondents’ socio-demographic and morbidity characteristics, as well as clinical and anthropometric parameters including body weight, height, blood pressure, neck, hip and waist circumference were collected. Data was captured using Epicollect5 and analyzed using SPSS version 20.0. *Results:* One-fourth (25.8%) of the respondents were at high risk of developing OSA. In terms of gender, 27.9% of the men and 23.8% of the women were at high risk for OSA. In univariate analyses, the risk of developing OSA was significantly associated with a history of diabetes mellitus, hypertension, dyslipidemia and gastro-esophageal reflux disease, weight, body mass index, neck, waist and hip circumference, waist–hip ratio, and systolic and diastolic blood pressure. Multivariate logistic regression analysis showed that a history of dyslipidemia (aOR, 95% CI = 2.34, 1.22–4.48), body mass index (aOR, 95% CI = 1.15, 1.06–1.22) and waist circumference (aOR, 95% CI = 1.10, 1.07–1.14) emerged as significant predictors of risk for OSA. *Conclusions:* A considerable proportion of NCD patients with easily detectable attributes are at risk of developing OSA, but still remain undiagnosed at a primary health care setting. The results obtained using MBQ in this study were comparable to studies performed using polysomnography. Dyslipidemia, body mass index and waist circumference were independent risk factors for predicting a risk of developing OSA. Prospective studies are needed to confirm whether a reduction in these risk factors could reduce the risk for OSA.

## 1. Introduction

Obstructive sleep apnoea (OSA) is a common disorder, characterized by repeated episodes of a complete or partial collapse of the upper airway (chiefly the oropharyngeal tract) at the time of sleep, with a subsequent reduction of the airflow [1]. In India, OSA prevalence varies from 9.3% to 13.7% [2,3]. The causes of developing OSA are multifactorial, comprising of a multifaceted interaction between anatomic, neuromuscular issues, along with an underlying genetic predisposition toward the disease [4]. The risk factors consist of snoring, a male gender, middle age, menopausal women, obesity and a range of craniofacial and oropharyngeal structures such as a bulky neck circumference, retro-or micrognazia, a nasal impediment, expended tonsils, and a low-lying soft palate [4,5].

As the ailment advancements, the sleepiness gradually becomes dangerous, resulting in reduced accomplishments at work and major job- and road-related mishaps [6]. Furthermore, many patients can suffer from cognitive and neurobehavioral impairment, an inability to concentrate, memory loss and mood variations such as irritability and depression. This further disrupts a person’s performance at work with significant results [7]. Sleep-related hypoxia has been linked with a systemic inflammation that may contribute to the commencement or quickening of the process of atherogenesis [8]. In addition, a significant metabolic deterioration arises in OSA independently from weight [6]. Insulin resistance, type 2 diabetes, and disturbed serum lipid parameters—broadly observed in patients with OSA—further increase in risk of cardiovascular illness [6,9,10,11].

Regrettably, numerous sufferers of OSA are undiagnosed [12]. This is likely because the standard measurement for diagnosis is overnight polysomnography (PSG) which is costly and not generally available [13]. Likewise, the attendant uneasiness of patients spending the whole night in sleep laboratories makes it rather impractical for large numbers of patients—whether as a diagnostic measurement tool or as a screening protocol [13].

A reasonable explanation for the foregoing difficulties in sleep study has been the advancement of questionnaires estimating sleep-related disorders and OSA [14]. Previous studies have utilized the standardized questionnaires like the Epworth sleepiness scale (ESS), the STOP (snoring, tiredness, observed apnea, high blood pressure) questionnaire, and the Cleveland sleep habits questionnaire (CSHQ) [13,14,15]. Still, the Berlin Questionnaire (BQ) remains the most extensively used screening questionnaire for OSA [15]. A Modified Berlin Questionnaire has been developed to suit Asian populations. Previously, the predictive value of the Modified Berlin Questionnaire for identifying the risk of developing OSA has been analyzed by using the receiver operating characteristic (ROC) curve, that resulted in a sensitivity of 85% and specificity of 95%. The positive predictive value was 96% and the negative predictive value was 82%. Questions about the symptoms demonstrated internal consistency (Cronbach α correlations 0.92–0.96) [16].

Research conducted in developed countries to illustrate and establish the risk factors linked with OSA reported that few of the risk factors are preventable [6,17,18]. Therefore, in this study, we focused on assessing and quantifying the risk factors associated with OSA and estimating the proportion of study participants who are at high risk of developing OSA, visiting an NCD clinic in a rural health training center attached to a tertiary care hospital of Puducherry, using the Modified Berlin Questionnaire. Additionally, we analyzed the cut-off value of waist circumference and BMI to predict the risk of developing OSA in the recruited subjects.

## 2. Materials and Methods

### 2.1. Study Setting

This facility-based cross-sectional study was undertaken in a peripheral health care setting from August 2018 to October 2018, among patients attending a non-communicable disease clinic. The rural health training centre (RHTC), Karikalampakkam, is one of the field practice areas of the department of community medicine—Indira Gandhi Medical College and Research Institute—located in the Union Territory of Puducherry, India. This RHTC caters for a population of around 37,000. Non-communicable disease clinics are conducted on a bi-weekly basis where new (i.e., referred from an out-patient department and an in-patient department) and old diabetic, hypertensive and cardiac cases come for a regular check-up as well as to collect their medication. Routinely, between 150 and 200 NCD patients are seen in a single NCD clinic day.

### 2.2. Inclusion Criteria

All Patients more than or equal to 18 years of age and less than or equal to 70 years attending NCD clinic for the first time during the study period were included in the study. 

### 2.3. Exclusion Criteria

Those with inadequate time (<6 h) in bed for sleeping (as adequate sleep is defined as between 6 and 8 h per night regularly) [19,20,21,22,23], extraneous sleep disruption (e.g., babies/children) and with shift work were excluded from the study.

### 2.4. Sample Size Estimation

Considering that the prevalence of OSA among the adult population is 13.7% [3], and taking the alpha error as 5% and absolute error of margin as 5%, the minimum sample size was calculated as 185. With the design effect of 2 for this systematic random sampling technique and the 10% non-response rate, we decided to take a minimum of 410 samples.

### 2.5. Sampling Technique

The study was conducted by interviewing every 3rd patient attending the NCD clinic following systematic random sampling.

### 2.6. Study Tool

The study participants were interviewed using a pre-tested semi-structured questionnaire, with questions on socio-demographic data, anthropometric data for assessing risk factors and the Modified Berlin questionnaire [16] for screening OSA. Physical parameters like height, weight, neck circumference, waist circumference, hip circumference, and BP were measured using standard procedures. A score of 2 or more positive categories indicates a high risk for OSA syndrome and only 1 or no positive category indicates a low risk for OSA syndrome.

### 2.7. Ethics

The data collection began after obtaining permission from the Institutional Ethics Committee located in Indira Gandhi Medical College & Research Institute, Kathirkamam, Puducherry, with its approval letter No. 17/IEC/IGMC/F-7/2017/28 dated 12 August 2017. Informed written consent was obtained from the study participants after explaining the objectives of the study.

### 2.8. Statistical Methods

The data was collected using epicollect5 and analyzed using Epi-info and SPSS version 20. The normality of the data distribution was assessed using the Shapiro–Wilk Test. Since the *p* value of the Shapiro–Wilk Test was greater than 0.05, the data distribution was normal and parametric tests were applied. The quantitative data was represented as mean and standard deviation and the qualitative data was described by proportions and percentages. The chi-squared test and Student *t*-test were used based on the data variables. A binomial logistic regression analysis was performed to determine the confounding factors and find the adjusted odds’ ratio. A receiver operating a characteristic curve analysis with the Youden index J statistic was performed to assess the optimal cut-off point for the significant anthropometric indices predicting risks of developing OSA. A *p* value of less than 0.05 was considered statistically significant.

## 3. Results

A total of 473 NCD patients participated in this study. The mean age of the study participants was 51 ± 11.34 years. The descriptive profile and univariate analyses of the study subjects are shown in Table 1. About 51% of the study subjects were males and so the male–female proportion is almost equal. The majority (67%) were less than 60 years old. Most of them (85%) were married. Almost 54% were sedentary workers, while the remainder were either moderate workers or heavy workers. A majority (75%) were non-smokers and non-drinkers of alcohol. About half of them were diabetic and hypertensive, and more than 15% had higher blood cholesterol and respiratory illness. Table 1 gives the association of socio-demographic and morbidity factors with risks of developing OSA. Among the study participants, 25.8% had a high risk of developing OSA based on the modified Berlin questionnaire criteria. From this study, it was found that a history of diabetes mellitus, hypertension, dyslipidemia and GERD were significantly associated with a greater risk of developing OSA.

The mean neck circumference, waist circumference and hip circumference of the study participants with a high risk of developing OSA was 37.26 ± 3.51 cm, 95.03 ± 8.49 cm and 104.47 ± 7.78 cm respectively. The mean waist–hip ratio among those with a high risk of developing OSA was 0.91, compared to a ratio of 0.89 among those with a low risk of developing OSA. The mean weight among those with a high risk of developing OSA (72.83 ± 10.75 kg) was a little higher than those with a low risk of developing OSA (63.83 ± 10.59 kg). As shown in Table 2, there was a statistically significant difference in the mean neck circumference, weight, body mass index, waist circumference, hip circumference, waist–hip ratio, systolic blood pressure and diastolic blood pressure between groups considered to be at a high risk and those considered to be at a low risk of developing OSA. In the univariate analyses, it was found that six out of seven anthropometric factors were significantly associated with a high risk of developing OSA.

In Table 3, the variables which were found to be significant from the univariate analyses (*p* < 0.05) in Table 1 and Table 2 were considered for binomial logistic regression analysis. However, on further analysis using binary logistic regression, only three risk factors were found to be associated significantly with a high risk of developing OSA. These were dyslipidemia (aOR 2.342, 95% CI 1.224–4.482), body mass index (aOR 1.147, 95% CI 1.075–1.223) and waist circumference (aOR 1.102, 95% CI 1.065–1.141), as presented in Table 3.

**Figure 1:**
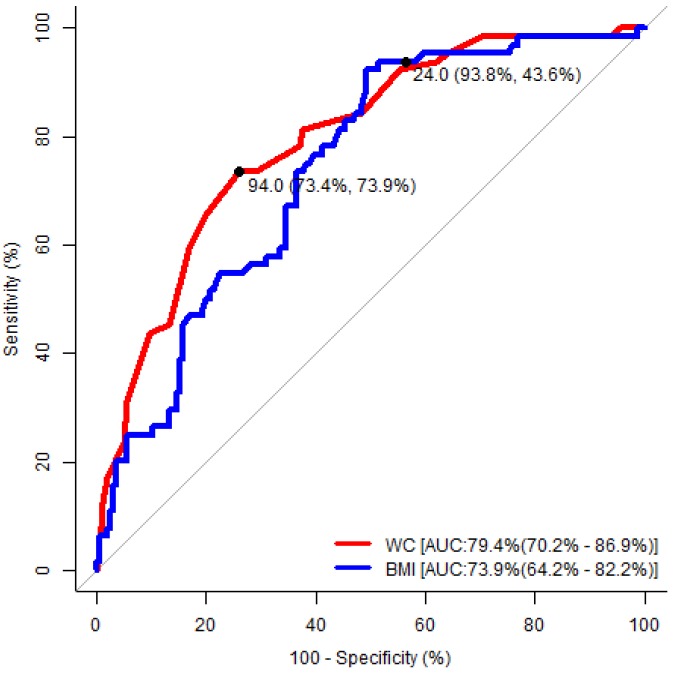
Receiver operating characteristic analysis to determine the optimal cut-off values of the significant anthropometric indices to predict the risk of developing obstructive sleep apnea (OSA) in males. WC: waist circumference; BMI: body mass index; AUC: area under the curve.

**Figure 2:**
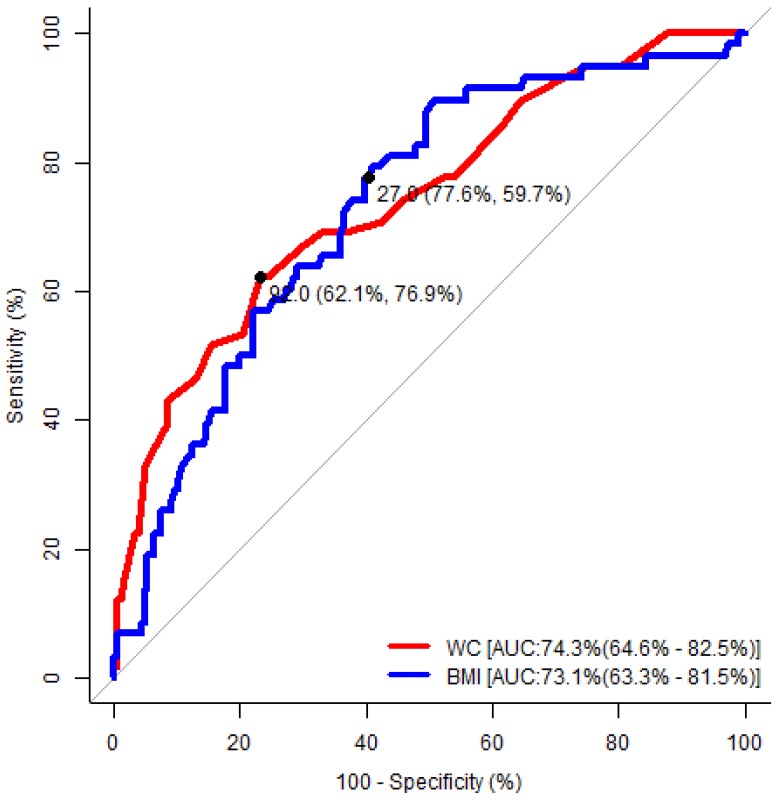
Receiver operating characteristic analysis to determine the optimal cut-off values of the significant anthropometric indices to predict the risk of developing obstructive sleep apnea (OSA) in females. WC: waist circumference; BMI: body mass index; AUC: area under the curve.

As depicted in Table 4, the area under the ROC curve was statistically significant in terms of waist circumference and body mass index, and the cut-off value had a high level of predictive accuracy for detecting the risk of developing OSA in both genders. In Figure 1, the values 94.0 cm for waist circumference (sensitivity, 73%; specificity, 74%), and 24.0 kg/m^2^ for body mass index (sensitivity, 94%; specificity, 44%) represented the optimum cut-off for males. In Figure 2, the values of 92 cm for waist circumference (sensitivity, 62%; specificity, 77%), and 27 kg/m^2^ for body mass index (sensitivity, 78%; specificity, 60%) represented the optimum cut-off for females.

## 4. Discussion

This facility-based study was conducted among NCD clinic attendees in a rural health training center under a medical college in Puducherry, using a Modified Berlin questionnaire which is a widely used, validated, reliable and simple tool for predicting the risk of developing OSA [11,12]. The proportion of patients attending the NCD clinic who were at a high risk of developing OSA was found to be 25.8% in this study. Viswanathan et al. documented that 23.65% of the study subjects attending diabetic clinic in Chennai had OSA [24]. Though this study utilized the Apnea–Hypopnea Index (AHI) to determine OSA, the present questionnaire-based study result matches with the above findings. Rashid et al. reported that 19.4% of the primary care clinic attendees in Malaysia were at a high risk of developing OSA when assessed using the BQ [25]. The lower prevalence in that study could be due to the fact that all of the patients attending the hospital were randomly assessed, whereas the present study focused only on the NCD clinic attendees. The present study observed that males (27.9%) were at a higher risk of developing OSA compared to females (23.8%). Similarly, a higher male preponderance for developing OSA has been documented by different researchers [24,26].

Despite the biologic plausibility of an association between smoking and the development of OSA, and the high prevalence of both the disorders, there is no adequate evidence to establish a significant relationship clinically [27]. This finding from the systematic review agrees with our study results. Simou et al. stated in their meta-analysis that people who consumed alcohol were about 25% more likely to suffer from OSA [28]. Consumption of alcohol increases the risk of developing OSA by reducing genioglossal muscle tone, leading to upper airway collapse and increased upper airway resistance [28]. The present study exhibited an association, though not one which was statistically significant. The reason could be that their treatment for the non-communicable disease may have prevented them from consuming alcohol and smoking.

Huang et al. identified a bidirectional association between OSA and diabetes mellitus in a large population-based study [29]. A similar association has been observed in the present study in the univariate analyses. Recurrent catecholamine production due to recurrent hypoxemia in OSA can impair glucose tolerance, reduce insulin sensitivity and cause type 2 diabetes mellitus [29]. Regarding diabetes mellitus and incident OSA, plausible factors like insulin resistance, leptin resistance, elevated systemic inflammatory metabolites and oxidative stress could reduce the airway response to hypercapnia, impair the neuromechanical control of airways and weaken the upper airway respiratory muscles—predisposing a person to developing OSA [29]. 

In a systematic review, Hou et al. reported that OSA and hypertension had a significant association [30], which corroborates the present study’s findings in the univariate analyses. Intermittent hypoxia in recurrent OSA can upregulate the sympathetic nervous system and cause sustained hypertension [30,31].

Kim et al. ascertained that esophagogastroduodenoscopy-proven GERD was significantly associated with OSA, which substantiates our study’s results. A recurrent upper airway narrowing among OSA patients during sleep could result in negative intra-thoracic pressure during inspiration [32]. The increased intrathoracic pressure might facilitate acid reflux into the esophagus, which finally leads to GERD [32]. GERD could deteriorate OSA by aggravating the airway resistance by causing posterior laryngitis [32].

The present study observed a strong association between dyslipidemia and OSA, which corroborates the findings which have been stated by Adedayo et al. in their systematic review [33]. Gündüz et al. observed a significant association between higher total cholesterol, higher LDL-cholesterol, elevated triglycerides, lower HDL-cholesterol and OSA [34]. Recurrent hypoxia during sleep in OSA patients could generate stearoyl-coenzyme A desaturase-1 and oxygen free radicals, leading to lipid peroxidation and eventually, dyslipidemia [33].

Kang HH et al. conducted a study among patients attending sleep clinics in Korea and found that the adjusted odds’ ratio and 95% CI for body mass index, neck circumference and waist circumference were 1.364 (1.22–1.53), 1.414 (1.24–1.62) and 1.114 (1.07–1.16) respectively [35]. The present study identified a similar risk in the anthropometric indices, except for neck circumference, as this study was conducted in non-communicable disease clinic. In a study which was conducted among the Turkish population, Soylu et al. found that BMI values over 28.93 kg/m^2^ in males and over 27.77 kg/m^2^ in females increased the risk of developing OSA [36]. Kang HH et al. found, in a study which was conducted among the Korean population, that BMI values over 24.95 kg/m^2^ in males and over 23.05 kg/m^2^ in females increased the risk of developing OSA [35]. The cut-off value for BMI being a risk factor for the development of OSA was over 24.00 kg/m^2^ in males and over 27.00 kg/m^2^ in females in the present study. The waist circumference cut-off for predicting the risks of developing OSA mentioned by Kang HH et al. was >105 cm for males and >101 cm female [35], whereas the present study delivered figures of >94 cm for males and >92 cm for females. The reason for these variations in the cut-off points could be the regional and hormonal influence in determining these factors. Subramanian S et al. established a gender difference concerning the risk factors for developing OSA where females had a higher BMI, higher hip circumference, lower waist–hip ratio, thin neck circumference and similar waist circumference compared to males. The present study also found a gender difference which was documented in Figure 1 and Figure 2, where a high BMI and similar waist circumference were observed among females compared to males as the cut-off to identify the risk of developing OSA [37].

This study has some limitations which must be discussed. First, because the current study was a cross-sectional study, the temporal association could not be assessed. Second, the predictive performance of modified BQ for the risk categorization of developing OSA, was not comparable to gold standard overnight polysomnography. This BQ has the highest sensitivity and specificity among the available tools for assessing the risk for OSA and is also a valid and reliable screening tool to be used in resource constraint settings. Third, the study subjects were taken from the NCD clinic and so generalizations had to be discussed based on these settings. Since non-communicable diseases have been escalating in India [38], the present study could show the magnitude and determinant factors of this neglected NCD.

## 5. Conclusions

OSA was determined as a common disorder among non-communicable disease patients, as it was observed in 27.9% and 23.8% of males and females, respectively. Dyslipidemia, body mass index and waist circumference were independent risk factors for predicting the risk of developing OSA, based on multivariate regression analysis. In addition, the present study reported the cut-off values of body mass index and waist circumference that increase the risk for OSA.

## Figures and Tables

**Table 1:** Association of socio-demographic and morbidity factors with risks of developing obstructive sleep apnea (OSA) (*n* = 473).

Variable	High Risk for OSA (%)	Low Risk for OSA (%)	*p* Value ^#^	Unadjusted OR (95% CI)
Gender				
Male	64 (27.9)	165 (72.1)	0.299	1.244 (0.823–1.879)
Female	58 (23.8)	186 (76.2)		
Age				
</=60 years	84 (26.3)	235 (73.7)	0.700	1.091 (0.701–1.699)
>60 years	38 (24.7)	116 (75.3)		
Marital status				
Others (unmarried/widow/divorce)	20 (29.9)	47 (70.1)		
Married	102 (25.1)	304 (74.9)	0.413	1.268 (0.718–2.241)
Occupation				
Sedentary worker	66 (25.6)	192 (74.4)		
Moderate worker	47 (26.4)	131 (73.6)	0.847	0.958 (0.620–1.480) Ref
Heavy worker	9 (24.3)	28 (75.7)	0.870	1.069 (0.480–2.383) Ref
Smoking history				
Smoker	38 (31.9)	81 (68.1)	0.077	1.508 (0.955–2.381)
Non-smoker	84 (23.7)	270 (76.3)		
Alcohol history				
Drinker	36 (31.0)	80 (69.0)	0.137	1.418 (0.893–2.251)
Non-drinker	86 (24.1)	271 (75.9)		
History of diabetes mellitus				
Yes	75(32.3)	157 (67.7)	0.001	1.972 (1.294–3.004)
No	47 (19.5)	194 (80.5)		
History of hypertension				
Yes	83 (31.0)	185 (69.0)	0.003	1.910 (1.237–2.948)
No	39 (19.0)	166 (81.0)		
History of Dyslipidemia				
Yes	32(45.1)	39(54.9)	<0.001	2.844 (1.686–4.799)
No	90(22.4)	312(77.6)		
History of Respiratory illness *				
Yes	24(30.8)	54(69.2)	0.272	1.347 (0.791–2.294)
No	98(24.8)	297(75.2)		
History of GERD				
Yes	32(38.1)	52(61.9)	0.004	2.044 (1.241–3.369)
No	90(23.1)	299(76.9)		
History of Sore throat				
Yes	10(30.3)	23(69.7)	0.539	1.273 (0.588–2.758)
No	112(25.5)	328(74.5)		

* Asthma/COPD; ^#^ Chi-square test; OR: odds ratio; CI: confidence interval; OSA: obstructive sleep apnoea; GERD: gastro-esophageal reflux disease.

**Table 2:** Association of clinical and anthropometric factors with risk for OSA (*n* = 473).

Variable	High Risk for OSA (N = 122) (Mean ± SD)	Low Risk for OSA (N = 351) (Mean ± SD)	*p*-Value ^$^	Mean Difference (95% CI)
Neck circumference (cm)	37.26 (3.51)	36.18 (3.34)	0.003	1.080 (0.362–1.798)
Weight (kg)	72.83 (10.75)	63.83 (10.59)	<0.001	8.99 (6.77–11.21)
Height (cm)	159.20 (8.27)	158.08 (8.29)	0.198	1.12 (−0.59–2.84)
BMI (kg/sq.m)	28.81 (4.23)	25.57 (4.03)	<0.001	3.24 (2.37–4.11)
Waist circumference (cm)	95.03 (8.49)	86.62 (8.87)	<0.001	8.41 (6.63–10.19)
Hip circumference (cm)	104.47 (7.78)	97.40 (7.79)	<0.001	7.06 (5.45–8.67)
Systolic blood pressure (mmHg)	141.57 (14.86)	133.77 (15.75)	<0.001	7.81 (4.68–10.93)
Diastolic blood pressure (mmHg)	86.42 (9.00)	83.07 (10.49)	0.001	3.35 (1.40–5.30)
Waist Hip Ratio	0.911 (0.067)	0.892 (0.088)	0.015	0.019 (0.0037–0.0338)

^$^ Students *t* test; SD: standard deviation; CI: confidence interval; OSA: obstructive sleep apnoea; BMI: body mass index.

**Table 3:** Predictors of risk for OSA by binary logistic regression analysis (*n* = 473).

	B	S.E.	Wald	*p*-Value	Adjusted Odds Ratio (95% CI)
History of DM (No as reference)	0.380	0.252	2.274	0.132	1.462 (0.892–2.394)
History of Dyslipidemia (No as reference)	0.851	0.331	6.601	0.010	2.342 (1.224–4.482)
History of GERD (No as reference)	0.093	0.309	0.092	0.762	1.098 (0.600–2.010)
Neck circumference	0.017	0.037	0.210	0.647	1.017 (0.947–1.092)
Body mass index	0.137	0.033	17.344	0.000	1.147 (1.075–1.223)
Waist circumference	0.097	0.017	31.090	0.000	1.102 (1.065–1.141)
Systolic blood pressure	0.019	0.010	3.748	0.053	1.019 (1.000–1.038)
Diastolic blood pressure	−0.006	0.014	0.166	0.683	0.994 (0.968–1.021)
Constant	−16.670	2.248	54.985	0.000	0.000

OSA: obstructive sleep apnoea; B: estimated logit coefficient; SE: standard error of the coefficient; Wald: test statistic using chi-square test; CI: confidence interval; DM: diabetes mellitus; GERD: gastro-esophageal reflux disease.

**Table 4:** Cut-off values for waist circumference and body mass index to predict the risk of developing OSA in males and females.

	Waist Circumference		Body Mass Index	
	Male	Female	Male	Female
Area under the ROC curve (95% CI)	0.79 (0.70–0.87)	0.74 (0.65–0.83)	0.74 (0.64–0.82)	0.73 (0.63–0.82)
*p*-value	<0.001	<0.001	<0.001	<0.001
Cut-off value	94.0 cm	92.0 cm	24.0 kg/m^2^	27.0 kg/m^2^
Sensitivity	73%	62%	94%	78%
Specificity	74%	77%	44%	60%

ROC curve: receiver operating characteristic curve; CI: confidence interval.
